# Uterine Myoma—Umbilical Hernia—Amputation of the Thigh

**Published:** 1892-06

**Authors:** Thomas H. Manley

**Affiliations:** New York


ATLANTA
### MEDICAL AND SURGICAL JOURNAL.

VOL. IX. JUNE, 1892. NO. 4.

EDITED BY
LUTHER B. GRANDY, M. D., AND MILLER B. HUTCHINS, M. D.,
WITH THE CO-OPERATION OF
H. V. M. MILLER, M. D., LL. D., VIRGIL O. HARDON, M. D.,
FLOYD W. McRAE, M. D., AND L. P. KENNEDY, M. D.

### *CLINICAL LECTURE.*

### UTERINE MYOMA—UMBILICAL HERNIA—AMPUTATION OF THE THIGH.

*Delivered at the Surgical Clinic of the Harlem Hospital, New York.*

BY
THOMAS H. MANLEY, M. D.,
NEW YORK.

GENTLEMEN: You will remember, in the notice of this clinic, it was announced that among other operations which we would proceed with to-day was one of abdominal section, for the removal of an interstitial fibroid of the womb; an abdominal hysterectomy, so-called. Well, here is the patient before you; and, while we must congratulate her on her escape from the perilous ordeal which was before her, we are sorry to have disappointed you.

But, it seems to me, that the history of this case and the simple means by which she has been rapidly and radically relieved of her infirmity are by far of more practical value than would be any operation, which would involve the loss of blood and an extensive mutilation. Her history is briefly this: she is 43 years old, free from any organic disease, and had good general health until a little more than three years ago, when simultaneously with a dragging pain in the back and loins, she noticed an unusual fullness over the uterus rather toward the left side. She failed to see her physician concerning it for nearly two years; when it had so increased in volume as to greatly inconvenience her and aggravate her pains. At this time, and for some time before it, she was having a copious metrorrhagia, between the catamenial epochs, which greatly emaciated and exsanguinated her. Her medical attendant has very patiently and thoroughly employed all the modern remedies, local and constitutional. She derived no benefit from any, except electricity, which, after each séance, greatly eased the pain. However, it made no impression on the neoplasm or her exhausting hemorrhages. Hence, as her health was steadily giving away, and she was incapacitated from doing any work, she was willing to submit to anything which offered her a promise of cure. And her physician, Dr. Nivison, of this city, a highly skillful practitioner, decided that a hysterectomy was the surest means of effecting a cure, though when he handed the case over to me he requested me to use my own judgment and deal with it as I thought best. I shall not say anything about differential diagnosis here, but will ask your attention to the various methods employed, when we treat these uterine myomata, by direct surgical intervention. They, as you know, are chiefly of two kinds, viz., palliative and radical. Among the former is the removal of the ovaries and ligation of the uterine arteries. The latter are the removal of the uterus and tumor by the abdominal or sacral incisions; their total removal by morcellation, as instituted and first extensively practiced by Pèan, of Paris; and, thirdly, the removal of the myomatous mass down through the cervical canal without any serious injury to the uterus, a method first successfully instituted by myself, in America, for the evulsion of very large uterine neoplastic growths. After having successfully operated last year for the removal of a uterine myoma, taking away womb, ovaries and all, on a careful examination of the specimen, we found that the growth might have been readily removed through the vagina, as was a very large, supposed inoperable tumor of this description, removed by me three years ago. Now, the removal of the ovaries is not in itself free from danger, and they may be so bound down, or dragged under the uterus as to be beyond our reach. But, what is the worst of all, their removal may in no way influence the growth of the mass. Ligation of the uterine arteries, when the cervix is drawn far up into the pelvic cavity, is impracticable; while, if attainable, the compensating dilatation of the ovarian vessels, will in a short time, make up for the occluded uterine arteries. It is unnecessary to remind you that every sort of operation undertaken, with a view of removing a large adherent uterus and tumor, is attended with great danger to life; and with the most expert is full of difficulties, besides many may be even impracticable.

Hence, in this case, before we proceeded to do a hysterectomy we first essayed to relieve our patient of her tumor by the utero-vaginal passage.

The cervical canal was gradually but widely opened by the use of tents when two fingers could be introduced into the uterine cavity, Now, on the left side, the uterine wall was felt compactly occupied by a large granular mass in its center, with an extensive capsule, which by adhesive inflammation, was glued to the entire endometrium. It was a homogeneous mass and seemed to consist of mixed elements. Little further was done at the first sitting. After a few days dilatation, thorough irrigation and drainage having been kept up, she was again placed on the table, when with the use of the volsellum-forceps, the long scissors and the curette, every remnant was swept away. The result has been as you see, the uterus, which two weeks ago was as large as a cocoanut, is now of its normal size. There is no more pain, no more morphine eating, and to-morrow or the next day she will leave the hospital.

The next case which I wish to show you is one of incarcerated, umbilical hernia in a woman. She is forty years old, has had four children. She first noticed this shortly after the birth of her last child. She comes to us for relief, because for the past year she can do no work, without having symptoms of strangulation, and is compelled from time to time to take the bed and apply soothing applications over the inflamed protrusion. You will notice the hernial mass is large, doughy, and but partly reducible. This species of hernia is the only one that I am acquainted with, which appears ever to arise as a consequence of the paturient effort in women. Although these extrusions in this situation are commonly designated umbilical in fact, they rarely are so, as they usually commence to form in the linea alba immediately above or below the ring and after their emergence insinuate themselves under the thin umbilical scar. One anatomical peculiarity about them well to remember is that they have no sac.

In this case my aim will be to freely expose and detach the displaced viscera. If the omentum is present in excessive quantity, it will be ligated and cut off. After having returned the hernia I will secure a complete and entire peritoneal covering in it and then close in the overlying structures by the continuous silk suture. The remaining parts will be closed in with catgut, layer by layer. No drainage will be employed.

The next patient is a man, on whom we will perform an amputation of the thigh. As his case is quite a unique one and conveys many useful lessons, it may repay us for the time consumed to rehearse part of it. Six days ago, while adjusting a belt on a pulley, he was caught and dragged up over a swiftly revolving shaft, and thrown about twenty feet. When our ambulance surgeon, Dr. Arch. Dixon, saw him, he was in a state of collapse and he feared he would not reach the hospital alive. However, he slightly reacted. When I saw him, two hours after the accident, I discovered that he had a dislocation of both knee-joints; the condyles of each femur being driven downward posteriorly and the head of the tibia upward and forward. The crucial and posterior ligaments, with the popliteus muscle, were completely torn through. Beside, he had a compound fracture of the right humerus close to the axillary space on the same side, fracture of the shafts of five ribs, in their center.

The luxated bones were easily replaced, but, as their ligamentous connections were destroyed, on the least movement of the limb, they at once slipped out of place again. We found that the best position that we could place the knees in, and which gave the most comfort, was by having them semi-flexed under pillows. The superior fragment of the broken humerus was drawn far inward, by the unopposed pectoralis major muscle, while its sharp-pointed end threatened to pierce the integument. The fractured ribs were not much displaced. Singular to say, that nowithstanding the terrible violence which the body had sustained, he reacted well and the following morning was in a fair condition. Both lower extremities from the patella downward were cool and showed unmistakable evidence of injury to their vascular supply. When we bear in mind that the popliteal artery lies close to the inter-condyloid space, that its sheath is firmly retained in position by a dense fascia, and that it is all the more fixed by the large terminal branches, which it gives off here, we can the better understand how a complete luxation at the articulation is impossible without seriously compromising the integrity of this vessel.

We now, by the use of hot bottles, hoped to avert the necessity of amputation, by endeavoring to favor collateral circulation. We were partly successful on the right side, but indubitable signs of dissolution in its fellow appeared on the third day, and we should have amputated sooner had we consent to do so. Now you will notice that the limb has a pale and shrunken appearance and that there are many patches on the surface of the integument, which resemble so many eschars from burns. During the past twenty-four hours gangrene has rapidly advanced towards the body, so that now it has reached the middle of the thigh, hence, no doubt, the best course to pursue would be to do a hip-joint amputation, but his general condition is so feeble that I fear the immediate consequences; accordingly we will carry the amputation knife through the upper third. You will notice that the opposite limb, though warm, has a bloated and ominous appearance.

Now a word about flaps, in amputation, before we commence. The only safe guide to surgeons in flap-making is experience. Too much tissue is as bad as too little, for we will have a slough; though it is always best when we select, to cut freely, so that, if there is a redundancy, it can be later trimmed away. It is only in pathological conditions, arising from a constitututional origin, that we can safely apply the classic amputation of the text-books. In traumatisms they may be wholly ignored, for the end which a conscientious surgeon should always have in view, is not a handsome, but a strong and useful stump.

NOTE.—Patient operated on for hernia is making an excellent recovery. The man on whom the amputation was performed developed septicæmia and died three days subsequently.

